# Editorial

**Published:** 1873-06

**Authors:** 


### Editorial.

### DIAGNOSIS.

### II.

I have hinted at some of the difficulties to be met with in tracing symptoms to their causes in a former number of this journal. Before developing the differential diagnosis of special affections, it seems proper to offer a word on the sympathetic relations of the different parts of the organism.

The intricacy and obscurity of the numerous cross-lines of sympathy, which bind together the parts and functions of the body, declaring themselves with especial emphasis during disease, often prove very embarassing to the practitioner, and sometimes constitute his principal hindrance to a correct diagnosis. The files of our dental journals contain numerous illustrative cases.

The direct response which the teeth make, to changes in the blood and to states of nutrition, and the reverse action by which causes at work in the teeth, produce serious and startling, often intensely damaging effects, upon the blood, and other parts, are so obvious as scarcely to require mention.

If causes are set in motion in the nervous system, sympathetic action spreads, and not unfrequently falls into channels of communication leading to the dental organs, from which reflected waves of influence beat back upon the general system.

The mixed character of these effects, and the embarrassment which the practitioner who does not understand the sympathetic relations of the parts of the body, must meet with in attempting a diagnosis, serve to impress their importance.

In diagnosing a case it should be remembered that the animal body is an organic unit, whose parts are mutually dependent, and that by consequence disease in any one organ is liable to introduce morbid action into any other, or even many others. As an insignificant atom of dust may disturb the motions of every wheel in a watch, so a morbific agent introduced at any point in the organism, may seriously disturb or entirely arrest action in the entire machinery of the animal body.

It should now be called to mind that diseased action is transmitted through certain channels more readily than through others; that it is obedient to laws of sympathy and susceptibility on the part of organs to which it is transmitted; and that slight and transient causes may divert it to unaccutomed channels, and to unusual tissues and organs.

In diagnosing a case it is necessary to know something of these relations and their causes; otherwise I know not how we shall be able to trace the usually obvious symptoms to their often entirely obscure pathological causes, and form in the mind an accurate picture of the extent, location, and character of the disease.

In some instances the symptom brings with it the evidence of its distant origin, and the path backward may be easily traced. But more often the dependence of the part which makes the outcry and contains the only visible sign of disease, upon some other and undeclared territory of morbid action, is quite hidden. Neither our own nor the patient's senses can point it out to us. It is only to be approached by a consecutive chain of reasoning, on the basis of the laws of sympathy and relation between the parts of the animal body.

And no matter how well we are armed with a knowledge of these laws, we will often meet with the most embarrassing difficulties. The clue which we have been following by a careful logic, backward to the source of trouble, sometimes ends suddenly in a tract, entirely barren of evidence. It is obvious that he who best understands physiological and pathological phenomena, and can most skilfully trace their relations, will be best prepared to make a difficult diagnosis.

It is worthy of remark that while much has been written in a careless away about the sympathies of organs and parts with each other, but little has been done toward tracing the laws of this sympathy; until this is done our efforts at diagnosis must often be blind and misdirected.

We have illustrative cases on record. Our case-books and journals are full of cases indicating special extraordinary sympathies of the teeth and associate parts, with distant organic functions, and pathological conditions.

In this way the eye, the ear, the scalp, the neck, shoulders the stomach, liver, spinal cord, the limbs along with the functions of fœcal excretion, menstruation, gestation, and lactation, have been pointed out as sympathising at times with conditions of the teeth ; while eplepsy, diseases of the antrum, hysteria trismus, uterine affections, and several important nervous diseases, have been shown to possess unmistakable connections with their pathological states.

But for the most part the cases on which these deductions rest have not been carefully studied and recorded. Their history has been imperfectly given ; the general state of health in the patients has received little attention ; diathesis has been largely ignored ; and many facts which it would be exceedingly desirable to know have been ommitted.

I have in my mind some admirable exceptions, but they are rare in the extreme. It is evident that before we can have any very complete developement of those special forms of pathological sympathy, which must in part constitute the basis of diagnosis in our profession, we must learn how to observe and record cases.

Those already recorded are highly valuable, but might have been much more so. They have pointed out to us special sympathies, and in many instances have furnished hints toward general laws of sympathetic action. What important conclusions may be drawn from a careful classification and comparison of these materials remains to be seen. It is evident that the attainment of general principles must be facilitated by whatever of accurate observation the future may record for us.

In this article I have only aimed to throw out a few hints to put the young practioner upon the right method of diagnosis. Any accurate statement of the laws of that immensely complicated and intricate weft-work, which makes up the sympathetic relationship of tissues, organs and functions, belongs of course to the differential diagnosis of special diseases, and would be best made in connection with the phenomena of each.

**The Microscope and Microscopic Technology;** by Dr. Hendrich Frey; translated by Geo. R. Cutter, M. D., from the fourth and last German edition. Wm. Wood & Co., N. Y., 1872.

Since the republication in this country in 1868; of Beale's "How to work the Microscope," no book has appeared which is likely to prove so useful to the student of microscopy as this.

It is a volume of six hundred and fourteen pages, abundantly illustrated with well executed engravings, and done in a style of typography which is excellent.

The mechanism of the microscope and the use of its parts, with the most essential optical principles on which its powers depend, are clearly explained in the first 106 pages. This is done briefly and in general terms, but with sufficient completeness for the student. An exhaustive discussion of technology which would only confuse the student, is left for larger works, designed for professionals; it being more the design of this volume to familiarize the learner with methods of manipulation and the preparation and mounting of specimens; and, while making him a practical microscopist, to give him some special training in the investigation of the various normal and abnormal tissues and fluids of the body.

This last is indeed a special feature of the work. It deals less with the mineral and vegetable kingdoms and carries its investigations largely into the domain of animal life, giving 386 pages to the study of human tissues. There are chapters on "Blood, Lymph, Chyle, Mucus and Pus; on Epithelium, Nails and Hair;" on "Connective Tissue and Cartilage;" on "Muscles and Nerves;" on the "Respiratory Organs," etc., etc.

It is therefore fitted to serve as a Manual of Histology for the student or physician.

Valuable observations on methods of manipulation and minute directions for the preparation and examination of the different tissues are scattered through every chapter; so that, while the work of drilling the student in manipulation is constantly kept in view, his knowledge of histology is very much advanced. It gives an excellent preparation for the more elaborate and exhaustive volume of Dr. Strieker.

**A Manual** of Histology by Prof. Stricker, of Vienna, Austria, in co-operation with Thomas Meynert, F. Van Recklinghausen, Max Shultze, W. Waldeyer and others. Translated by Henry Power of London, and eight others of New York and Boston, with 431 illustrations. New York: William Wood & Co., 27 Great Jones street, 1872.

In this large volume of 1100 pages, we have by far the most important survey of histological science that has ever been published. And in saying this we do not in the least detract from the lofty honors of Kolliker. Prof. Stricker stands in the front rank of histologists. He has brought to his assistance a corps of specialists and experts whose investigations have been most extensive and profound in the departments to which they have devoted themselves.

The following are a few of the chapters with names of their authors:

General Methods of Investigation. by S. Stricker.
General Character of Cells, by S. Stricker.
The General Structure of the Nervous System, by Max Shultze.
The Heart, by F. Schmeiger Seidel.
The Blood Vessels, by C. J. Ekerth.
The Lymphatic System, by F. Van Recklinghausen.
The Spleen, by Wilhelm Muller.
The Salivary Glands, by E. F. W. Pfluger
Structure and development of Teeth, by W. Waldeyer.
Intestinal Canal, by C. Taldt.
The Testicle, by Van La Voulte St. George.

And thus the topics run through about fifty chapters and until all the tissues and secretions have been carefully examined.

Here is a manual which is not, like too many of our text books, made up of ill-chosen selections by a hired compiler. It has united in its pages a constellation of the brightest names in histology. Prof. Stricker has brought their labors into one volume, and into such relation as to make them cover the entire range of human histology.

There is no second-hand knowledge. The papers are all original and replete with the freshest observation. The works of the different writers, in fact, take the character of Monographs, and have been neither clipped nor retouched.

With all its merits the work has some defects. But one can hardly have a heart to draw attention to them in presence of so much excellence.

We could wish that Prof. Stricker in his introduction had given some account of the general routine of histological work instead of filling the whole space with a description of some special late methods. That the student can find the general routine of work in smaller books is hardly an excuse, since it is precisely in this complete work that we ought to find it.

The two chapters on the character of cells are perhaps unequaled by anything in the English language. And the same may be said of Max Shultze's exposition of the minute structure of the nervous system. It is admirable in both its matter and form, beyond criticism.

The paper by Ludwig on the Kidney is an artistic revelation of the structure of this organ.

Meynert's paper has wandered a little from the problems of histology to demonstrate the connection of all parts of the brain with each other. On a cursory examination, it is not easy to determine whether the embarrassing obscurities to be met with in this paper are due to the intricacies of the subject, or to a defective method of treatment.

But it is impossible in short space to criticise or even mention each of these generally admirable and exhaustive papers. The work, as it should be, could only be reviewed by an editorial corps of experts almost as large as that of the body of specialists who have produced it.

As a manual for the student of histology it is now the indispensable text book. Each subject being treated by a special investigation in that department, the work not only presents the science of histology in its full and rounded proportion, but it presents nearly all that is at this time known of the science.

We can heartily recommend this truly admirable manual to every student of human histology.

### PERSONAL.

Dr. H. L. Ambler, of Cleveland, a graduate of the Ohio Dental College started on an European trip June 1st inst. He will have a grand old time. We have a little fear that he may find inducements—professional that may prompt his return for sometime at least.

While we are most happy to be represented by such men as Dr. A., we can't afford to lose them from this country, but if they will go, we think that men will be raised up to take their places here.

### REMOVAL.

Dr. E. M. Morrison of Noblesville Ind. has removed to Des M oins Ind. Dr. Morrison for many years has been one of *the* men of the Dental profession in Indiana, and his removal will be quite a loss, not only to those who have been accustomed to avail themselves of his skill, but to the profession as well. He has been an active and prominent member of the Indiana State Dental Society from its organization.

### DENTAL.

### A NEW COLLEGE.

We have received the announcement of the Maryland Dental College of Baltimore City, which is to hold its first annual session, October 1st, 1873. Already, we have nine dental colleges in the United States, and with the opening of this one we shall have ten.

The course of instruction includes both Didactic and Clinical lectures from a corps of professors who are with one exception, engaged in the actual practice of Dentistry.

It is mentioned in the amendment that graduates of this institution will be received as second course student at the Washington University of Medicine.

All information may be obtained of the Dean R. R. Winder. No. 128 West Madison street.
Baltimore Md.

### DON'T ANÆSTHETIZE A FEMALE WITHOUT A WITNESS.

About twenty years ago, a dentist of Philadelphia was convicted of a foul crime, on the testimony of a female to whom he had administered chloroform for a dental operation. He was sentenced to a long term of imprisonment, and though subsequently pardoned, it was not till an incurable pulmonary disease had been developed by his confinement. There were serious questions as to his guilt, and we believe the opinion finally prevailed that the charge was unjust, and that the woman was the subject of a delusion, arising from the action of the anæsthetic on her brain. Recently, a similiar accusation has been brought against a dentist in England, and he has been held to bail for trial, on the testimony of the woman concerned, unconfirmed by other evidence. The occurrence of these, and other instances of similiar character, displays the impropriety of giving anesthetics to a female, under any circumstances, without the presence of a witness. This should be a settled rule of action, both by dentists and physicians. *Ex.*

### A TALE OF A NOSE.

The readers of our surgical works are doubtless familiar with those cases in which the nose is reported, after having been cut off, to have successfully replaced—in one instance, after a dog had run away with it. An interesting example of the reparative power of nature in such an injury, is recorded in last week's *Wiener Médizin*, *Wochenschr*, by Dr. Malfatti. On November 13, 1871, Lieutenant Preiser, while in barracks, had his nose cut off by a sabre, The wound passed through the middle of the cartilaginous part of the bridge of the nose, dividing the left alæ along its posterior third, the septum through its middle, and the left ala two lines behind the anterior angle of the nostril. The piece cut off was taken up from the ground where it lay, cleaned with cold water, and reapplied, being secured in its place by means of sutures. A week after the injury, a dry scab began to form on the left alæ, and extended in the course of three days over nearly the whole nose. Granulations gradually formed, healing went on steadily, and on February 1, 1872, careful examination was required to detect any trace of the injury.—*Brit. Med. Journal*, Dec. 21, 1873.

